# Research on Lettuce Canopy Image Processing Method Based on Hyperspectral Imaging Technology

**DOI:** 10.3390/plants13233403

**Published:** 2024-12-04

**Authors:** Chao Chen, Yue Jiang, Xiaoqing Zhu

**Affiliations:** Research Center of Fluid Machinery Engineering and Technology, Jiangsu University, Zhenjiang 212013, Chinaqiaoq202207@163.com (X.Z.)

**Keywords:** lettuce, moisture content, hyperspectral imaging, non-destructive detection, intensity correction, feature selection

## Abstract

For accurate segmentation of lettuce canopy images, dealing with uneven illumination and background interference, hyperspectral imaging technology was applied to capture images of lettuce from the rosette to nodule stages. The spectral ratio method was used to select the characteristic wavelengths, and the characteristic wavelength images were denoised and image fused before being processed by filtering and threshold segmentation. To verify the accuracy of this segmentation method, the manual segmentation method and the segmentation method used in this study were compared, and the area overlap degree (AOM) and misclassification rate (ME) were used as criteria to evaluate the segmentation results. The results showed that the segmentation effect was the best when 553.8 nm, 702.5 nm and 731.3 nm were selected as the characteristic wavelengths of lettuce for the spectral ratio method, with an AOM of 0.9526 and an ME of 0.0477. Both have a variance of less than 0.01 and have the best stability. Hyperspectral imaging technology combined with multi-wavelength image and multi-threshold segmentation can achieve accurate segmentation of lettuce canopy images.

## 1. Introduction

Crop hyperspectral images not only contain crop information but also background information such as that of soil [1] Background information can affect the accuracy of the crop physiological information prediction model, and it is necessary to segment the hyperspectral image to remove the background image elements and purify the spectral data of the crop. Hyperspectral images contain both image information and spectral information, resulting in a very large amount of data in hyperspectral images, which makes the analysis and calculation difficult and the processing speed slow; therefore, the characteristic wavelength images should be extracted and combined with image processing methods to extract the target region [2].

In terms of extracting crop leaves and canopy pixels, some scholars extracted characteristic wavelength images for analysis by comparing the differences in the spectral curves of ground objects. Yue et al. [3] compared the spectral curve difference between longan leaves and soil background, selected the image at 519 nm as the feature image, and successfully segmented the whole longan leaf area by automatic binarization to remove the background area. Zhang [4] used partial least squares regression and difference spectrum analysis to extract four characteristic wavelength images from cotton hyperspectral images and combined wavelength image arithmetic and threshold segmentation methods to achieve the segmentation of ground cover with a recognition rate of 91.07%. Zhu [5] extracted a single wavelength image at 700 nm and used the large difference in gray scale between the green leaves and white background, as well as threshold segmentation, to remove the background region and extract the tomato leaf region with a good segmentation effect. Zhang et al. [6] selected an image at a wavelength of 420 nm using the adaptive band selection method and segmented the hyperspectral image of lettuce using the micrologic method to obtain the complete lettuce leaf region with satisfactory segmentation results. All the above studies extracted the characteristic wavelength images and used the threshold segmentation method to segment the leaf and background regions. However, these studies have not fully utilized the advantages of hyperspectral imaging data, and these should be studied in depth for multiple background areas. In terms of pests and diseases, surface damage and impurity detection, hyperspectral image segmentation techniques are more mature and provide references for leaf and canopy extraction studies. Wang et al. [7] used principal component analysis to extract feature wavelengths from hyperspectral images of maize root stubble rows and segmented images by single threshold segmentation and median filtering methods for feature images, respectively, with a segmentation accuracy of 91.85%. Tian et al. [8] analyzed the hyperspectral images of corn borer holes and corn, selected the feature images with the best combination of single and dual wavelengths by using the blending distance as the criterion, and segmented the images by combining image processing methods to determine whether there was an infestation, with a correct rate of 96%. When segmenting hyperspectral images of leaves and canopy, most scholars use single wavelength image segmentation to extract crop areas. The information in the hyperspectral image data is lower, which needs to be studied in depth when facing complex backgrounds.

In this paper, the potted soil lettuce collected indoors was taken as the research object, and hyperspectral information of the lettuce canopy with different water contents and hyperspectral images of the lettuce canopy with accurate segmentation were obtained. A multi-threshold segmentation method combined with multi-wavelength images was proposed to eliminate complex background information and extract the complete lettuce canopy image. Aimed at the problems of uneven illumination and background interference of lettuce canopy, the spectral ratio method was used to extract the spectral data, with the largest difference between the leaf and background as the characteristic wavelength. The median filtering method was used to denoise the characteristic wavelength image, and the denoised image was fused. The characteristic wavelength images, fusion images and images after principal component analysis under single and double thresholds were segmented, respectively. The segmentation results were evaluated according to the area overlap and misclassification rate, and the optimal segmentation method was selected.

## 2. Materials and Methods

### 2.1. Experimental Design

The lettuce variety used in the trial was Italian year-round mossy lettuce, cultivated in potted soil culture from 30 September 2021 to 3 January 2022 in the sprinkler irrigation laboratory at Jiangsu University. We put 3~5 seeds in each pot, waited until they grew to ‘five leaves and one heart’, cleaned up each pot, and left only one seedling with similar growth. Six water control treatments [9,10] were selected to irrigate them, each corresponding to 25 potted plants, using drip arrows (SLD109 + SLD012 type, Guangzhou Shunlv Sprinkler Equipment Co., Ltd., Guangzhou, China) for irrigation. The six treatments were as follows: the planned wetting layer was set to be 15 cm, and the soil moisture content was maintained at 40–50%, 50−60%, 60−70%, 70−80%, 80−90%, 90−100% of the field water holding capacity, respectively. A soil moisture sensor (HM-WSY type, Shandong Hengmei Technology Electronic Co., Ltd., Guangzhou, China) was used to measure the soil moisture content under each treatment every day. When the soil moisture content reached the lower limit, irrigation was performed until the soil moisture content reached the upper limit.

### 2.2. Hyperspectral Image Acquisition

**Figure 1:**
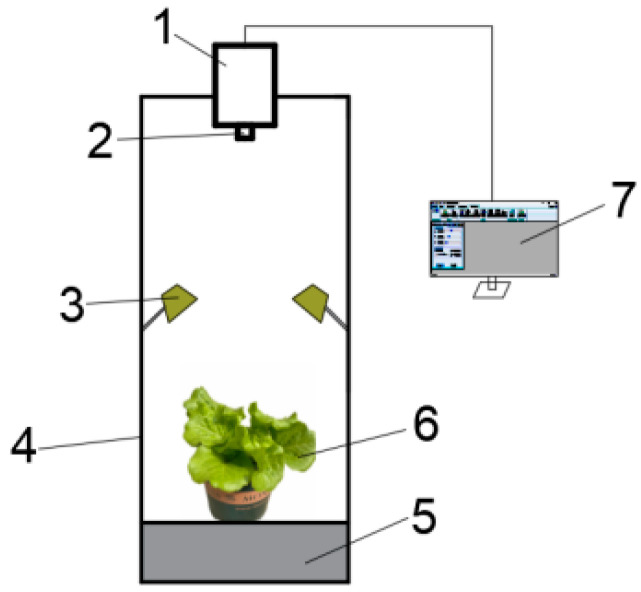
Hyperspectral imaging system. 1. Hyperspectral imager. 2. Lens. 3. Light source. 4. Light-shielding test bench. 5. Stage. 6. Lettuce sample. 7. Monitor.

**Figure 2:**
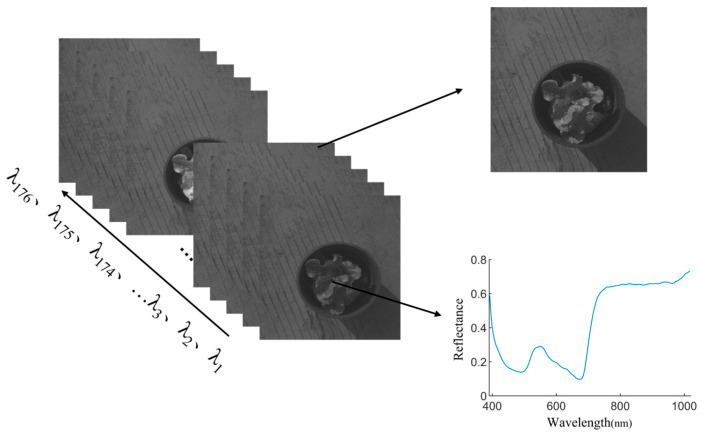
A hyperspectral image data block containing single wavelength image and single-pixel spectral information.

To reduce the influence of natural light on spectral data, experiments were conducted in a dimly lit environment. The test bench is shown in Figure 1. We preheated and debugged the instrument before collecting hyperspectral images. We used the imager to extract the hyperspectral image data of the standard whiteboard, observed the spectral image data of the whiteboard, and adjusted the objective lens distance and exposure time, so that the whiteboard was in the center of the image and filled the image area, ensuring that the light intensity of each area of the whiteboard was uniform. After repeated debugging, the lens-to-whiteboard distance was set to 75 cm and the exposure time of the camera was set to 2.5 ms [11]. The imager was used to obtain the hyperspectral image data of the black and white grid, and the full spectral curve of the black and white grid was observed to adjust the focal length. When the spectral curve was of the step type, the focusing was successful [12]. When collecting hyperspectral images, the top of the lettuce canopy was placed at the same height as the above whiteboard, and the position was horizontally adjusted to fill the camera perspective. The data were acquired by Spectra VIEW, Version 2.9.2.43, and each sample was acquired three times and the average was taken as the individual sample data. The single sample hyperspectral image data were a data matrix of 1101 × 960 × 176, as shown in Figure 1. The data block shown in Figure 2 contains 176 grayscale images. The grayscale value of the grayscale image is the spectral value of the sample, and the full-band spectral data of any pixel can be seen in the image.

To eliminate the influence of light intensity and dark current noise in the camera on spectral image quality, black and white calibration of spectral data was performed [13]. Under the experimental conditions, the hyperspectral image data W of the standard white plate were acquired. We covered the CCD camera lens to obtain the hyperspectral image data B of the blackboard and correct the original spectral image in black and white according to Formula (1):

(1) $$R=\frac{I-B}{W-B}$$

In the formula, I is the original spectral data of lettuce canopy; R is the corrected lettuce canopy spectral data. B is the data of a standard black background panel; W is the data of a standard white calibration panel.

In this study, MATLAB 2018a software was used for the black and white correction, as well as the subsequent mathematical statistics algorithm and digital image processing technology.

### 2.3. Extraction of Lettuce Canopy Region Segmentation Based on Feature Image

#### 2.3.1. Image Enhancement

Image enhancement refers to highlighting useful information in an image while attenuating or removing certain useless information with the aim of making the image more suitable for observation and analysis. Median filtering [14] is a typical image enhancement technique that preserves image detail features while removing noise such as image pulses and spike interference. Median filtering is a typical image enhancement technique that preserves image detail features while removing noise such as image pulses and spike interference. Median filtering usually uses a sliding window of an odd number of image points and takes the median value of the grayscale in the neighborhood instead of the grayscale value of the central image element [15]. In this paper, a median filter with a template size of 3 × 3 was used to process the characteristic image of lettuce wavelength.

#### 2.3.2. Image Fusion

The essence of image fusion is to synthesize multiple wavelength images into one image through some mathematical algorithms, which is conducive to maximizing the extraction of favorable information in each wavelength image and improving the accuracy of image segmentation. It is widely used in image processing. At present, fusion methods such as wavelength image arithmetic operation and principal component analysis are widely used in hyperspectral image processing. The wavelength arithmetic operation is a supervised method, which is conducive to extracting small target areas [16,17]. In this paper, images of three wavelengths are processed using the arithmetic mean. The expression (2) is as follows:

(2) IMG=IMG_1+IMG_2+IMG_3

In the formula, IMG_1, IMG_2 and IMG_3 are three wavelength images, respectively, and IMG is the fused image.

#### 2.3.3. Image Segmentation

Image segmentation is a method of dividing the whole area of an image into several disjoints and multiple regions with different meanings, and the features within each region are similar in nature, with the aim of making the segmented region easy to identify and analyze [18,19]. Considering that there are many elements in the lettuce feature image; the reflection characteristics of leaves, shadow leaves and the background are inconsistent; and the fact that they cannot be classified into one category, multi-threshold segmentation should be adopted [20,21].

The maximum interclasses variance method [22], also known as the OSTU method, is an adaptive thresholding segmentation method. The principle of the method is as follows: let the gray level of the target image be L and set a threshold t to divide the image into two parts: the target area $T_{1}$ and the background area $T_{2}$; $P_{T_{1}}\left(t\right)$ and $P_{T_{2}}\left(t\right)$ are the proportion of pixel points in $T_{1}$ and $T_{2}$ to the total number of pixels, respectively, and the corresponding gray level averages are $\mu_{T_{1}}\left(t\right)$ and $\mu_{T_{2}}\left(t\right)$, which are calculated by the following formula.

(3) $$P_{T_{1}}\left(t\right)=\sum_{i=0}^{t}p_{i},P_{T_{2}}\left(t\right)=\sum_{j=t+1}^{L-1}p_{j}$$

(4) $$\mu_{T_{1}}\left(t\right)=\sum_{i=0}^{t}\frac{ip_{i}}{P_{T_{1}}\left(t\right)},\mu_{T_{2}}\left(t\right)=\sum_{j=t+1}^{L-1}\frac{ip_{j}}{P_{T_{2}}\left(t\right)}$$

In the formula, $p_{i}$ is the proportion of the number of pixels with a gray value of i to the total number. Calculating the mean value $\mu_{0}$ of the entire image pixel, the formula is as follows:

(5) $$\mu_{0}=\sum_{j=t+1}^{L-1}ip_{i}$$

The variance between classes $\sigma^{2}$ between the target region $T_{1}$ and the background region $T_{2}$ is

(6) $$\sigma^{2}=P_{T_{1}}{\left(\mu_{T_{1}}-\mu_{0}\right)}^{2}+P_{T_{2}}{\left(\mu_{T_{2}}-\mu_{0}\right)}^{2}$$

Set *t* in the range of [0, L − 1] and calculate the between-class variance $\sigma^{2}$. When $\sigma^{2}$ is taken as the maximum, t is the best segmentation threshold. 

When extending to multi-class segmentation, the threshold vector is set as {*t*_1, *t*_2, …, *t*, *k*}, where 1 ≤ *k* < L; the target image can be divided into *k* + 1 classes, and the probability $P_{T_{i}}$ and the mean $\mu_{T_{i}}$ of each class can be found according to Equations (3)–(5), respectively. Then, the variance $\sigma^{2}$ of class *k* + 1 can be obtained according to Equation (7):

(7) $$\sigma^{2}=\sum_{i=1}^{k+1}P_{T_{i}}\left(\mu_{T_{i}}-\mu_{0}\right)$$

The threshold vector is traversed in the range [0, L − 1], and the corresponding threshold vector is optimal when the variance $\sigma^{2}$ between multiple classes is maximum.

#### 2.3.4. Segmentation Accuracy Evaluation Method

To evaluate the segmentation accuracy of lettuce canopy, the original lettuce image was segmented by the manual method and compared with the above image segmentation results, and the area overlap measure (AOM) and misclassified error (ME) were used as evaluation indexes to measure the segmentation performance, respectively [23].

Among them, the AOM is used to analyze the deviation between the result area of the segmentation algorithm and the manually segmented area, which is calculated as follows.

(8) $$AOM=\frac{Area\left\{S_{1}\cap S_{2}\right\}}{Area\left\{S_{1}\cup S_{2}\right\}}$$

In the formula, $S_{1}$ is expressed as the region extracted by the algorithm; $S_{2}$ represents the artificially segmented region; and $Area\left\{\;\right\}$ is the number of pixels in the region. The larger the value of AOM is, the better the segmentation effect is, and when AOM is 1, the segmentation effect is the best. ME represents the ratio of the number of misclassified pixels to the total number of manually segmented pixels, and the number of misclassified pixels is the sum of under-segmented regions and over-segmented regions. The calculation formula is as follows:

(9) $$ME=\frac{Area\left\{S_{1}\cup S_{2}\right\}-Area\left\{S_{1}\cap S_{2}\right\}}{Area\left\{S_{1}\right\}}$$

The smaller the value of ME is, the better the segmentation effect is. When ME is 0, the segmentation effect is the best.

## 3. Results and Discussion

### 3.1. Selection of the Optimal Splitting Wavelength

In the lettuce canopy image segmentation based on color and gray areas, the greater the color or gray difference between the lettuce leaf area and the background area, the more favorable it is for extracting the lettuce canopy area [24]. The spectral acquisition range of the spectral imager is 400~1000 nm, so in this band range, the mean pixel value of the background and target area is calculated. The larger the mean difference is, the greater the difference between the background and the leaf is. We analyzed the RGB image extracted from the hyperspectral imagery and the grayscale images corresponding to the seven spectral bands with the largest differences in reflectivity between the background and the canopy.

**Figure 3:**
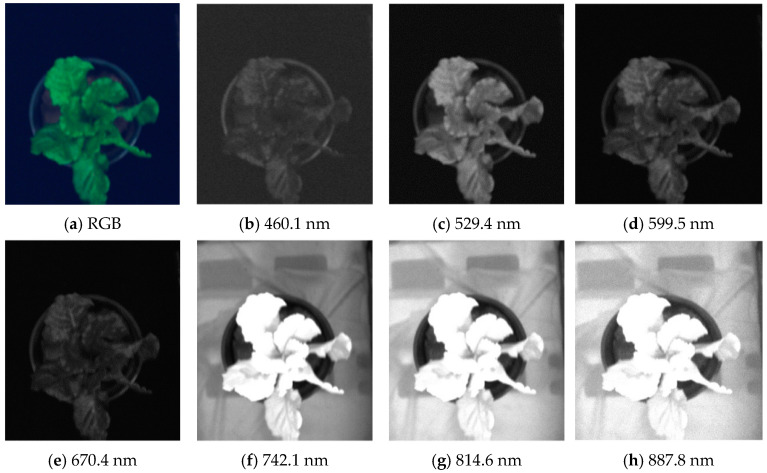
Lettuce canopy images at different wavelengths.

As the original lettuce canopy image at each wavelength is dark, it is not easy to observe the background noise of the image, so the image is enhanced, as shown in Figure 3. Figure 3a displays an RGB image derived from the hyperspectral imagery, while Figure 3b–h shows the grayscale images of the hyperspectral imagery at wavelengths of 460 nm, 529.4 nm, 599.5 nm, 670.4 nm, 742 nm, 814.6 nm, and 887.8 nm, respectively.

From Figure 3, it can be found that the grayscale values of lettuce canopy leaves and each background region are different at different wavelengths, and the images can be segmented according to these differences. In the 529.4 nm and 742.1 nm wavelength images, the difference between the leaf and the background is more obvious, and the leaf reflectance is stronger in the wavelength range of 742.1 to 887.8 nm, but there are also some shadow leaves with lower reflectance. Since the greater contrast between lettuce leaves and background is more favorable to extract the lettuce canopy region, the ratio of reflectance between the lettuce region and each background region was calculated, and the image with the greatest color difference was identified from 176 wavelength images as the optimal wavelength image for lettuce sample segmentation. The average spectral reflectance of each region was calculated as follows:

(10) $$f_{i}=\frac{1}{Ni}\sum_{j=1}^{N_{i}}F_{ij}$$

In the formula, $f_{i}$ is the average full spectrum vector of the *i*-th region; $F_{ij}$ is the full spectral vector of the *j*-th pixel in the *i*-th region, *i* = 1, 2, 3, 4, 5; and *N*1~*N*5 are the number of pixels in the area of lettuce normal leaves, shaded leaves, soil, flowerpots and the black stage.

**Figure 4:**
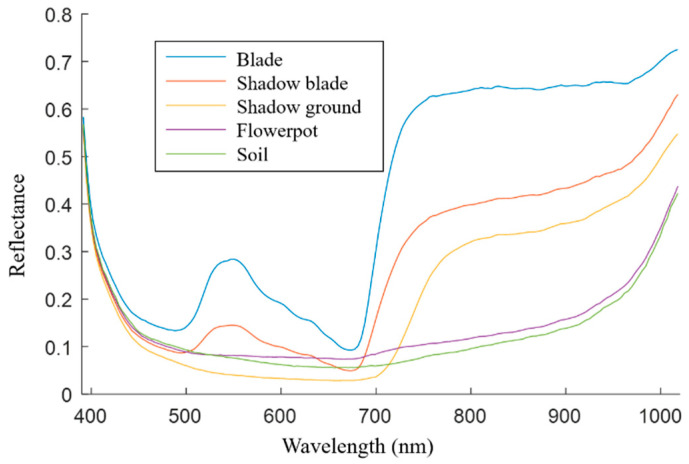
Spectral reflectance of different areas in canopy image.

Figure 4 shows the average spectral reflectance curves of normal leaves, shaded leaves and background regions of lettuce canopy. It can be seen from the figure that the spectral reflectance of lettuce leaves and background regions differed at different wavelength bands. In the band of 400~500 nm, the difference in spectral reflectance between the leaf area and soil and background area was not obvious. In the 400~500 nm band, the difference in spectral reflectance between the leaf region and the soil and background regions was not significant. In the 500~600 nm band, the spectral reflectance of the leaf area was slightly larger than that of the background area. In the 700–1000 nm band, the spectral reflectance of the leaf region was significantly higher than that of the soil and background regions.

**Figure 5:**
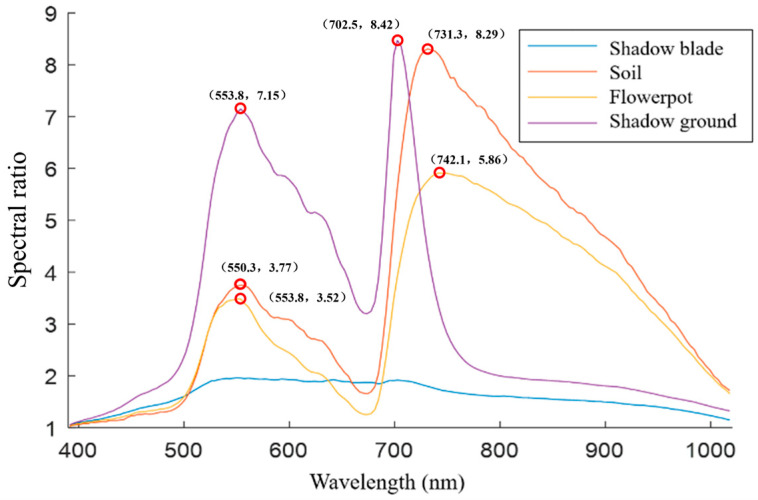
Spectral ratio of leaves to other regions.

The reflectance ratio curves for normal leaves, shadowed leaves, and background areas across the entire spectral range are depicted in Figure 5. Upon examining the figure, it is evident that each background spectral ratio curve reaches its maximum values at two specific wavelengths: 553.8 nm with a ratio of 7.15 and 702.5 nm with a ratio of 8.43 for one curve; 550.3 nm with a ratio of 3.77 and 731.3 nm with a ratio of 8.29 for another; and 553.8 nm with a ratio of 3.52 and 742.5 nm with a ratio of 5.87 for the third. These observations indicate that there are notable differences between the spectral signatures of the background and the leaves at these particular wavelengths. At the wavelengths of 553.8 nm, 702.5 nm, and 731.3 nm, the reflectivity differences among shaded leaves, soil, and pots are pronounced and more representative, with no interference among their corresponding spectral reflectance, which facilitates improved segmentation classification accuracy. Consequently, images at these three wavelengths were selected for subsequent image analysis.

### 3.2. Image Enhancement and Fusion

**Figure 6:**
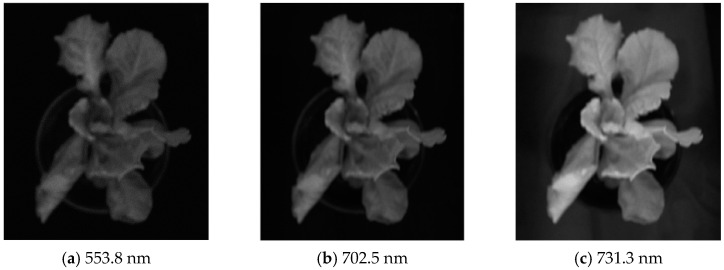
Characteristic wavelength image.

As illustrated in Figure 6, grayscale images at wavelengths of 553.8 nm, 702.5 nm, and 731.3 nm were extracted and served as the feature wavelength images. Firstly, median filtering was performed on the feature image to remove the local noise in the image. Then, a variety of fusion methods were used to compare the difference between the background gray level and the leaf gray level. It was found that the sum of the three images can make the average gray values of the three backgrounds close to each other. The three backgrounds can be classified into one category, and the average gray levels were 0.0681, 0.0890 and 0.0701, respectively, while the average gray levels of normal leaves and shadow leaves were 0.3983 and 0.2135. It was found that the gray values of the three types of regions were significantly different, so this method was selected for image fusion.

**Figure 7:**
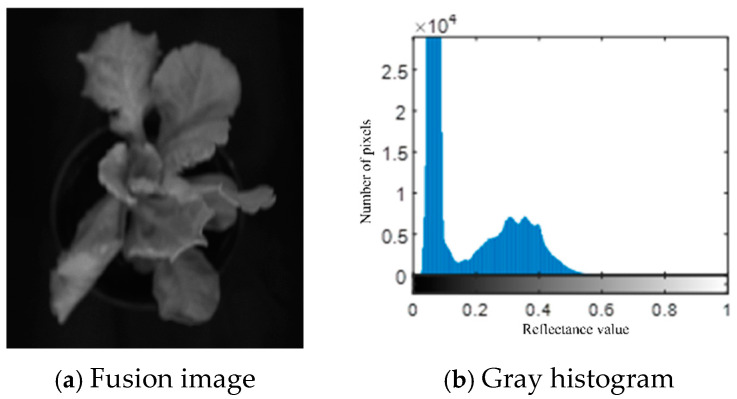
3 Band mean image and corresponding histogram.

The fused image is shown in Figure 7a, and its gray histogram is shown in Figure 7b. From the histogram, it can be seen that the lettuce canopy image presents a bimodal characteristic, with a certain three-peak trend. The first peak is significantly different from the second.

### 3.3. Canopy Region Segmentation of Lettuce in Hyperspectral Image

**Figure 8:**
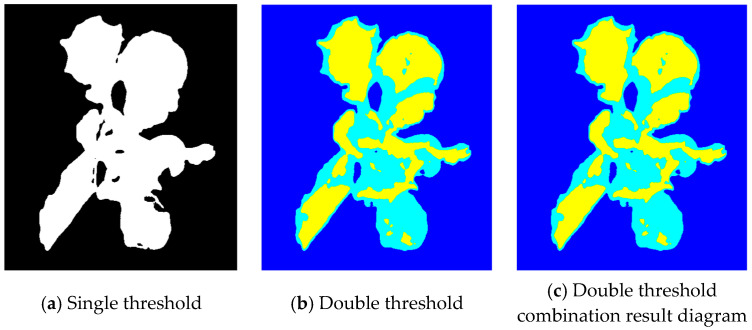
Single threshold and double threshold image segmentation results.

The fusion image was segmented by single and double thresholds, respectively. The results are shown in Figure 8. Figure 8a is the result of single threshold segmentation. Among them, the overexposed and normal leaves are segmented normally, but the darker areas in the middle and the edge areas of the lettuce are under-segmented, and there are less over-segmented areas. Figure 8b is the result of double threshold segmentation. The background, shadow leaves, and normal leaves are divided into three categories, and the darker edge parts and shadow leaves are classified into the second category. The overall segmentation effect is better. Figure 8c shows the merged results of normal and shadowed leaf image elements; the segmentation results are better and can be used for masking hyperspectral images.

**Figure 9:**
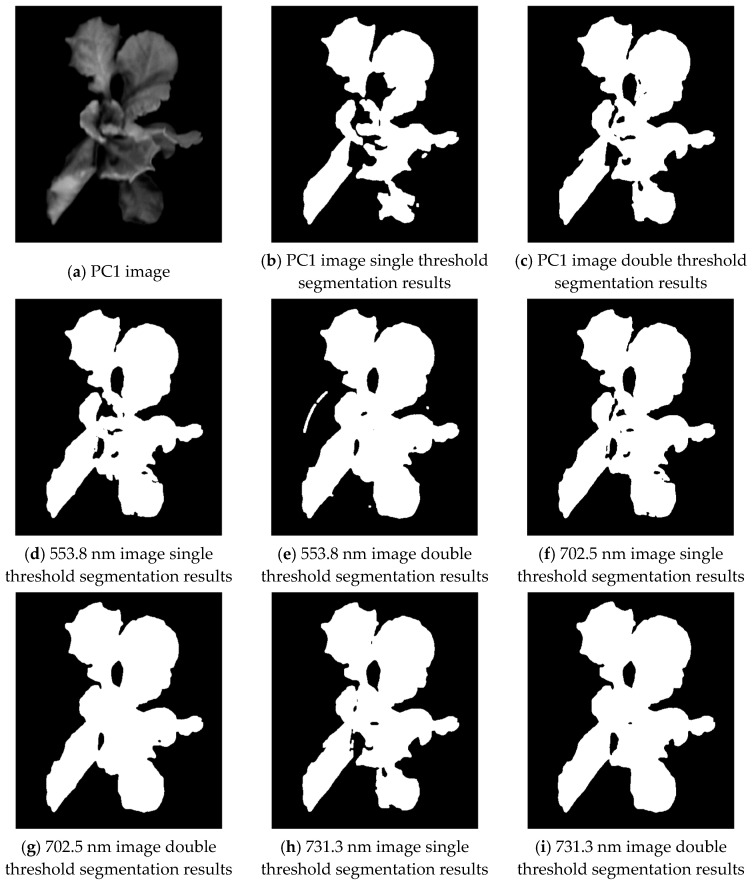
Image segmentation results of single threshold and double threshold.

PCA [25] (principal component analysis) is a commonly used technique in the field of image processing, particularly suitable for dealing with image data containing a large amount of redundancy and noise. This redundancy and noise information can significantly interfere with image analysis. PCA effectively addresses this challenge by removing redundant information and highlighting major features. During the PCA process, the original image data are projected onto principal components (PCs) that are sorted according to their contribution to data variation. Among them, PC1, as the first principal component, represents the direction of maximum variation in the dataset and embodies the main features of the data. Therefore, PC1 can accurately capture the core variability information in the dataset, providing strong support for image processing and significantly improving the accuracy and efficiency of the processing. For comparative analysis with the fusion method proposed in this paper, we applied PCA to process three wavelength images and selected the PC1 image for segmentation processing. The PC1 image and three single wavelength images are subjected to median filtering to remove noise, and then four images are segmented using the single threshold and double threshold, respectively, and the random noise is removed by open operation. The segmentation results are shown in Figure 9.

It is observed that the over-segmentation in the threshold segmentation results of the PC1 image is serious, and many shaded leaf regions are mis-segmented as background regions. The double threshold segmentation results of the three characteristic wavelength images were better but also produced some mis-segmentation. To better evaluate the segmentation effects of multiple methods, 12 lettuces hyperspectral image datapoints were selected for the experimental segmentation study, and the accuracy and stability of the segmentation algorithms were evaluated by the mean and variance in AOM and ME; the segmentation results are shown in Table 1.

From the analysis of the single- and double threshold segmentation results, the AOM of the single threshold segmentation of the 553.8 nm image is higher than the double threshold, and the ME is lower than the double threshold, probably because the gray values of each region are similar and not easily distinguishable. In the rest of the wavelength images, the gray values of normal leaf, shadow leaf, and background regions are different; therefore, the double threshold segmentation results are better than the single threshold segmentation results. Analyzed from the perspective of feature images, the AOM of the PC1 image is lower than the AOM value of the remaining images; the ME value is higher than the remaining images, because the background of each lettuce canopy sample image is more complex, and the principal component analysis algorithm is easily disturbed by noise points, so the segmentation effect is poor and less stable. The double threshold segmentation of the fused images had the best effect and the highest stability, with mean values of AOM and ME of 0.9526 and 0.0477, respectively, and corresponding variances of 0.0111 and 0.0110, respectively, indicating that the three regions were well distinguished and the algorithm could segment the lettuce canopy leaves better.

## 4. Discussion

Previous research has exhaustively explored the application of various classical image segmentation techniques in crop canopy segmentation tasks, clearly revealing their unique strengths and limitations. We conducted a comprehensive evaluation of classical methods such as fixed thresholding [26], Otsu’s method [27], Canny edge detection [28], and the watershed algorithm [29] in terms of their ability to accurately segment the foreground (i.e., crop canopy) while minimizing background interference. These evaluations were then compared with the results of the fusion-based dual-threshold segmentation method proposed in this paper.

The fixed thresholding method achieved an AOM value of 0.7762, indicating a high degree of overlap between the segmented canopy and the actual canopy. However, the ME value of 0.0700 also revealed certain errors in pixel allocation. In contrast, Otsu’s method showed a slight improvement in the AOM value, indicating enhanced foreground segmentation capability, while the ME value dropped significantly to 0.0151, highlighting the significant advantage of Otsu’s method in accurately distinguishing between the foreground and background.

The Canny edge detection method exhibited excellent performance in canopy boundary detection, with an AOM value as high as 0.8980. However, its ME value of 0.2458 was unusually high, which may be due to over-segmentation or sensitivity to noise resulting in significant pixel misclassification. This further emphasizes the need to carefully balance high boundary detection classification accuracy and high pixel misclassification error when using Canny edge detection for canopy segmentation.

Although the watershed algorithm exhibited a relatively low AOM value and performed less well compared to other methods, its ME value of 0.0163 indicated minimal pixel classification errors. This may be related to the watershed algorithm’s tendency to produce over-segmentation when processing images with complex topological structures, leading to confusion between the foreground and background. However, within the correctly segmented regions, the method had relatively few pixel misallocations.

Notably, the fusion-based dual-threshold segmentation method surpassed all traditional methods in terms of AOM, demonstrating exceptional foreground segmentation classification accuracy. This is primarily attributed to the enhanced contrast and clarity provided by the fusion process, enabling more precise threshold setting. However, in terms of background elimination, the dual-threshold method was slightly inferior to Otsu’s method and the watershed algorithm, meaning that while it excelled in identifying the canopy, it may still face challenges in accurately delineating the background, potentially leading to background contamination in the segmented images.

Overall, the fusion-based dual-threshold segmentation method has demonstrated unique advantages in crop canopy segmentation, particularly in terms of foreground segmentation classification accuracy, surpassing many traditional methods. This discovery provides new perspectives and ideas for the development of image segmentation techniques, especially in the current era where deep learning segmentation techniques are becoming increasingly popular.

Despite the significant achievements of deep learning algorithms in image segmentation, their complex model structures and substantial computational resource requirements pose certain challenges [30]. In contrast, the fusion-based dual-threshold segmentation method stands out in application scenarios with limited resources or high real-time requirements due to its simplicity and ease of implementation. Furthermore, this method does not rely on extensive training data, making it potentially more adaptable and flexible in certain specific domains or emerging scenarios.

In crop canopy segmentation, the fusion-based dual-threshold segmentation method not only significantly improves foreground segmentation classification accuracy but also effectively reduces background interference, providing more accurate data support for subsequent crop growth monitoring, pest and disease diagnosis, etc. The successful application of this technology further validates the huge potential and significant value of traditional image segmentation methods in specific scenarios, while also providing new insights for the optimization and improvement of deep learning algorithms.

Looking ahead, with the continuous advancement of image processing and computer vision technologies, the fusion-based dual-threshold segmentation method is expected to be combined with other advanced technologies to jointly develop more efficient and accurate crop canopy segmentation solutions. At the same time, to address its limitations in background elimination, researchers can further explore the integration of additional post-processing steps or the development of more complex segmentation algorithms to continuously enhance segmentation effectiveness and expand its application value. In today’s world where deep learning segmentation techniques boast global popularity [31], the fusion-based dual-threshold segmentation method not only offers a promising alternative but also injects new vitality and directions for thought in the development of image segmentation techniques.

## 5. Conclusions

In this study, the lettuce canopy image and background image were segmented by hyperspectral imaging technology. The research conclusions are as follows:

(1) Wavelengths with large differences between lettuce leaves and background regions were extracted by the spectral ratio method and were 553.8 nm and 731.3 nm, 550.3 nm and 742.1 nm, 553.8 nm and 702.5 nm, respectively. The wavelengths with similar characteristics were removed by the principle of band correlation, and the three wavelengths of 553.8 nm, 702.5 nm and 731.3 nm were finally extracted as the characteristic wavelengths with the largest difference between the background and the leaf spectrum.
(2) The characteristic wavelength image was processed by median smoothing to remove local noise. The filtered image was processed by the band algorithm for image fusion. The average gray levels of the three backgrounds were 0.0681, 0.0890 and 0.0701, respectively, while the average gray levels of the normal leaves and shadowed leaves were 0.3983 and 0.2135, respectively. There was a significant difference in gray values between background and leaves. This method was used for image fusion to improve the accuracy of the image segmentation. To facilitate comparison with the fusion method in this study, three wavelength images were processed using PCA.
(3) In this study, three characteristic wavelength images, fusion images and PC1 images obtained by principal component analysis were segmented by single and double threshold methods, and the segmentation results were evaluated by area overlap (AOM) and misclassification rate (ME). In addition to the 553.8 nm image, the AOM of the single threshold segmentation was higher than the double threshold, and the ME was lower than the double threshold. In the remaining wavelength images, the double threshold segmentation results were better than the single threshold segmentation results. After PCA processing, the AOM of the image was lower than that of the other images, the ME value was higher than that of the other images, and the segmentation result was not good. The results showed that the multi-threshold segmentation of multi-wavelength fusion images was the best. The average values of AOM and ME were 0.9526 and 0.0477, respectively, and the corresponding variances were 0.0111 and 0.0110, respectively, which indicate the accurate segmentation of lettuce canopy images.

## Figures and Tables

**Table 1:** Image segmentation performance evaluation.

Segmentation Object	Segmentation Method	AOM				ME			
		Maximum Value	Minimum Value	Average Value	Variance	Maximum Value	Minimum Value	Average Value	Variance
553.8 nm Image	Single Threshold	0.9250	0.8500	0.8875	0.0229	0.1501	0.0777	0.1132	0.0230
	Double Threshold	0.9513	0.6375	0.8753	0.0892	0.5658	0.0492	0.1529	0.1447
702.5 nm Image	Single Threshold	0.9278	0.8653	0.9043	0.0210	0.1347	0.0723	0.0961	0.0208
	Double Threshold	0.9658	0.7119	0.9304	0.0695	0.4023	0.0346	0.0798	0.1021
731.3 nm Image	Single Threshold	0.9368	0.8315	0.8962	0.0320	0.1685	0.0633	0.1038	0.0320
	Double Threshold	0.9668	0.9187	0.9464	0.0159	0.0813	0.0336	0.0538	0.0158
Fusion Image	Single Threshold	0.9392	0.8456	0.9032	0.0277	0.154	0.069	0.0970	0.0276
	Double Threshold	0.9687	0.9322	0.9526	0.0111	0.067	0.031	0.0477	0.0110
PC1 Image	Single Threshold	0.8950	0.7439	0.8209	0.0442	0.256	0.105	0.1792	0.0442
	Double Threshold	0.9502	0.8466	0.8954	0.0317	0.153	0.049	0.1047	0.0316

Note: area overlap degree (AOM); misclassification rate (ME); PCA segmentation (PC1).

## Data Availability

Data are contained within the article.
